# Lithium as a Potential Neuroprotective Strategy in Glaucoma: Mechanisms and Therapeutic Perspectives

**DOI:** 10.3390/biom16071062

**Published:** 2026-07-20

**Authors:** Alessio Martucci, Giulia Napoleoni, Annagrazia Adornetto, Francesco Aiello, Raffaele Mancino, Massimo Cesareo, Giacinto Bagetta, Carlo Nucci, Enrico Romano, Rossella Russo

**Affiliations:** 1 Ophthalmology Unit, Department of Experimental Medicine, University of Rome “Tor Vergata”, Via Montpellier 1, 00133 Rome, Italy; martucci@med.uniroma2.it (A.M.); giulia.napoleoni@ptvonline.it (G.N.); francesco.aiello@ptvonline.it (F.A.); mancino@med.uniroma2.it (R.M.); massimo.cesareo@uniroma2.it (M.C.); nucci@med.uniroma2.it (C.N.); 2 Department of Pharmacy, Health and Nutritional Sciences, Section of Preclinical and Translational Pharmacology, University of Calabria, 87036 Rende, Italy; annagrazia.adornetto@unical.it (A.A.); giacinto.bagetta@unical.it (G.B.); 3 Department of Sense Organs, Sapienza University of Rome, 00185 Rome, Italy; enrico.romano@uniroma1.it

**Keywords:** retinal ganglion cell degeneration, neuroprotection, repurposing, IOP-independent therapy, optic neuropathy

## Abstract

Glaucoma is a major global health concern, identified as the foremost cause of irreversible blindness, affecting nearly 95 million individuals. It is characterized by the progressive degeneration of retinal ganglion cells (RGCs), leading to significant vision-related disabilities and an extensive socio-economic impact. The concept that glaucoma should be viewed not solely as an ocular condition but also as a neurodegenerative disorder, sharing pathophysiological features with diseases like Alzheimer’s and Parkinson’s, is now widely accepted. This review examines the convergence of molecular mechanisms, including the roles of amyloid precursor proteins and neuroinflammation, that contribute to RGC loss. Notably, lithium, traditionally used as a mood stabilizer, has emerged as a potential neuroprotective agent for the treatment of Alzheimer’s disease. In light of the common neurodegenerative mechanisms linking glaucoma with central neurodegenerative diseases, here, we review the current evidence supporting lithium’s therapeutic potential in glaucoma, emphasizing the need for further clinical studies to determine its effectiveness in preserving optic nerve health and improving patient outcomes.

## 1. Introduction

Glaucoma is the leading cause of irreversible blindness and comprises a heterogeneous group of optic neuropathies characterized by the progressive loss of retinal ganglion cells (RGCs), axonal degeneration and visual field (VF) impairment [1,2,3,4,5].

Due to the slow progression of vision loss, the frequent asymmetry between eyes, and the occurrence of compensatory neurological mechanisms, patients often remain undiagnosed until advanced stages of the disease [6]. Nevertheless, VF defects significantly affect personal independence and quality of life, impairing both functional and psychosocial domains [1,3,7].

Risk factors for glaucoma include advanced age, male sex, myopia, family history, smoking, race, systemic hypotension and hypertension, vasospasm, the use of systemic or topical steroids, migraine, obstructive sleep apnea syndrome, and elevated intraocular pressure (IOP) [8].

Among these factors, IOP remains the only modifiable target of currently available glaucoma therapies [5]. Indeed, elevated IOP can directly compress the lamina cribrosa, leading to axonal deformation and impaired axoplasmic flow, ischemic stress of the optic nerve head (ONH) and consequent RGC injury [9].

However, elevated IOP does not always coexist with the occurrence of glaucoma, and a subset of patients experience progressive VF deterioration despite having normal or adequately controlled IOP. These observations imply that additional pathogenic mechanisms, other than IOP elevation, such as vascular dysregulation, mitochondrial impairment, excitotoxicity, and neuroinflammation may contribute to the pathogenesis of the disease [10].

Glaucoma is increasingly recognized as a neurodegenerative optic neuropathy involving both ocular and central nervous system components sharing several molecular determinants and hallmarks with neurodegenerative central disorders such as Alzheimer’s disease (AD) and Parkinson’s disease (PD) [11,12]. This would point to the possibility of identifying common therapeutic targets and shared therapeutic approaches.

Despite encouraging results in experimental models, virtually all neuroprotective therapies investigated for glaucoma have failed to demonstrate convincing efficacy in clinical trials. Several factors probably contribute to this translational gap. Animal models generally reproduce only selected aspects of glaucomatous neurodegeneration and often involve acute injury paradigms that differ substantially from the chronic, multifactorial progression observed in patients. Moreover, glaucoma is a heterogeneous disease involving multiple pathogenic mechanisms, including mechanical stress, vascular dysregulation, oxidative stress, mitochondrial dysfunction, neuroinflammation, and age-related susceptibility. Therapeutic strategies directed against a single molecular target have therefore produced limited clinical benefit. Additional challenges include difficulties in identifying sensitive clinical endpoints, the slow rate of disease progression requiring prolonged follow-up, interindividual variability in disease course, and uncertainty regarding the optimal timing of neuroprotective intervention [7].

Despite this, given its long history of clinical use, well-characterized pharmacological profile, and well-documented neuroprotective effects in several neurodegenerative diseases that share pathogenic mechanisms with glaucoma, the repurposing of lithium for glaucoma may represent a promising therapeutic strategy, while also providing valuable insights into novel molecular targets and fostering the development of new neuroprotective therapies for optic neuropathies.

Therefore, the purpose of this review is to provide a comprehensive and critical outline of lithium-based therapy in glaucoma by integrating current preclinical and clinical evidence, exploring lithium’s neuroprotective, anti-apoptotic, and RGC-preserving effects, and identifying gaps in knowledge to guide future research and potential clinical applications.

## 2. Literature Search Strategy

A comprehensive literature search was conducted to identify experimental and clinical studies investigating the role of lithium in glaucoma and related neurodegenerative disorders. Electronic searches were performed in PubMed/MEDLINE, Scopus, and Web of Science for articles published from database inception through June 2026. The search strategy combined Medical Subject Headings (MeSH) and free-text terms, including “lithium”, “glaucoma”, “retinal ganglion cells”, “optic neuropathy”, “neuroprotection”, “GSK-3β”, “Wnt/β-catenin”, “oxidative stress”, “neuroinflammation”, “autophagy”, “mitochondrial dysfunction”, “Alzheimer’s disease”, “Parkinson’s disease”, and “retinal degeneration”. Boolean operators (“AND”, “OR”) were used to optimize the search strategy.

Original research articles, clinical studies, observational studies, systematic reviews, and relevant narrative reviews published in English were considered eligible. Additional publications were identified through manual screening of reference lists of key articles. Conference abstracts, editorials, letters without original data, duplicate publications, non-English articles, and studies lacking relevance to the neuroprotective mechanisms of lithium or glaucoma were excluded.

Titles and abstracts were independently screened by the authors for relevance, followed by full-text evaluation of potentially eligible articles. Studies were selected based on their scientific quality and relevance to the objectives of this review, with particular emphasis on investigations addressing lithium-mediated neuroprotective mechanisms, RGC survival, glaucoma pathophysiology, and shared molecular pathways with other neurodegenerative diseases. The final selection included studies considered most relevant to the aims of this narrative review.

## 3. Glaucoma as a Neurodegenerative Disease: Shared Pathogenetic Mechanisms with Central Disorders

Glaucoma is increasingly recognized as a neurodegenerative disorder affecting both the eye and the central visual pathways. Neuroimaging and histopathological studies have demonstrated atrophy of the lateral geniculate nucleus, while advanced diffusion imaging has revealed structural alterations extending beyond the primary visual cortex to regions involved in motor and cognitive processing. These cerebral changes correlate with retinal nerve fiber layer (RNFL) thickness and macular ganglion cell integrity, supporting a continuum of neurodegeneration from the retina to the brain [13,14,15,16]. This pattern is consistent with trans-synaptic degeneration, whereby neuronal injury spreads to synaptically connected cells, progressively compromising the entire visual pathway [17].

A hallmark of neurodegenerative diseases is the selective vulnerability of specific neuronal populations [7,18]. In AD, early degeneration affects the entorhinal cortex, hippocampus, nucleus basalis of Meynert, and locus coeruleus, whereas PD primarily targets neuromelanin-containing dopaminergic neurons of the substantia nigra pars compacta. Similarly, glaucoma preferentially affects large-projecting RGCs, particularly magnocellular/parasol-type cells [19,20,21]. This selective neuronal loss is driven by shared pathogenic mechanisms, including mitochondrial dysfunction, oxidative and endoplasmic reticulum stress, impaired axonal transport, glutamate excitotoxicity, neuroinflammation, abnormal protein processing, altered proteostasis and vascular dysregulation [22].

Experimental and clinical studies have demonstrated the presence of β-amyloid (Aβ), phosphorylated tau, and α-synuclein in glaucomatous retinas, suggesting that molecular hallmarks traditionally associated with AD and PD are also present in glaucoma [23,24,25]. In experimental models of chronic ocular hypertension, abnormal amyloid precursor protein processing leads to Aβ accumulation within the optic nerve (ON) and RGC layer [26,27]. Aβ deposition promotes activation of microglia and astrocytes, triggering neuroinflammatory responses that contribute to retinal degeneration [28,29]. Consistent with these findings, increased concentrations of several AD-related biomarkers, including apolipoproteins A1, CIII, and E, transthyretin, and α2-macroglobulin, have been detected in the aqueous humor of patients with primary open-angle glaucoma [30,31].

Neuroinflammation represents a central mechanism linking glaucoma with other neurodegenerative disorders [32]. Activated microglia and reactive astrocytes chronically release pro-inflammatory cytokines, nitric oxide, and complement factors that exacerbate neuronal stress, axonal degeneration, and RGC loss [33,34,35]. Within the trabecular meshwork, these inflammatory mediators promote extracellular matrix remodeling, cytoskeletal dysfunction, cellular senescence, oxidative stress, and NF-κB activation, ultimately impairing aqueous humor outflow and contributing to elevated IOP [36].

Recent studies have also identified dysregulation of the WNT/β-catenin signaling pathway, characterized by increased glycogen synthase kinase-3β (GSK-3β) activity, in both the retina and trabecular meshwork of glaucomatous eyes [37]. Because WNT/β-catenin signaling regulates neuronal survival, oxidative stress responses, synaptic stability, and cellular repair, its suppression promotes mitochondrial dysfunction, inflammation, and increased susceptibility of RGCs to apoptosis while also contributing to trabecular meshwork dysfunction and impaired IOP homeostasis [38,39,40,41,42]. Accordingly, pharmacological inhibition of GSK-3β has emerged as a promising neuroprotective strategy capable of reducing neuroinflammation, stabilizing β-catenin, and enhancing neuronal survival [24].

Autophagy, a lysosome-mediated pathway responsible for the degradation and recycling of cellular components, is another key mechanism implicated in glaucoma [43,44,45]. Physiologically, autophagy helps trabecular meshwork cells adapt to mechanical stress generated by aqueous humor dynamics, thereby preserving tissue integrity and outflow function [46,47]. However, under chronic mechanical stress, autophagy may become dysregulated and shift from a protective to a maladaptive process, contributing to both trabecular meshwork dysfunction and RGC apoptosis [47,48,49]. Similar impairment of autophagic flux occurs in AD and other neurodegenerative disorders, where defective clearance of misfolded proteins promotes accumulation of Aβ and hyperphosphorylated tau [50,51]. Reduced expression of the autophagy regulator beclin-1 further contributes to this process, whereas experimental enhancement of autophagy restores lysosomal function, facilitates clearance of toxic protein aggregates, and attenuates neurodegeneration, highlighting autophagy modulation as a shared therapeutic target across neurodegenerative diseases [52,53,54].

## 4. Lithium as a Neuroprotective Modulator

Lithium, a classical mood stabilizer long used in the treatment of bipolar disorder, has increasingly been recognized for its pleiotropic neuroprotective properties. Beyond its psychiatric applications, lithium has demonstrated therapeutic potential in several neurodegenerative disorders, including PD, Huntington’s disease (HD), and AD [24]. Lithium is involved in fundamental physiological processes, including neuronal signaling, synaptic plasticity, myelin integrity, neurodevelopment, and maintenance of brain homeostasis [55]. Accordingly, disruption of lithium homeostasis may weaken these mechanisms and facilitate neurodegenerative processes [56]. Consistent with this concept, epidemiological studies suggest that long-term lithium exposure is associated with a reduced incidence of dementia [57,58], while numerous preclinical studies have demonstrated lithium-mediated attenuation of neuronal loss [59,60,61].

Preclinical studies in rodent models and human neurons have shown that chronic lithium administration upregulates key neuroprotective proteins, including B-cell lymphoma 2 (Bcl-2) and brain-derived neurotrophic factor (BDNF), thereby promoting neuronal survival and synaptic plasticity [62]. One of lithium’s principal molecular targets is GSK-3β, a serine/threonine kinase that regulates tau phosphorylation and multiple signaling pathways involved in neuronal survival [24]. By inhibiting GSK-3β, lithium reduces tau hyperphosphorylation and neurofibrillary tangle formation, while also modulating oxidative stress, neuroinflammation, apoptosis, and autophagy [24,63,64].

At the cellular level, lithium regulates gene expression in neurons, astrocytes, microglia, and oligodendrocytes. Lithium deficiency induces transcriptomic changes resembling those observed in AD, including downregulation of synaptic and myelin-related genes together with activation of inflammatory and neurodegenerative pathways [55].

Recent evidence has further implicated endogenous lithium homeostasis in AD pathogenesis [56]. Analysis of post-mortem brain tissue demonstrated significantly reduced lithium concentrations in the prefrontal cortex of individuals with mild cognitive impairment and AD compared with cognitively normal controls. Moreover, lithium was found to accumulate within amyloid-β plaques, potentially reducing its bioavailability in surrounding brain tissue. Lower brain lithium concentrations were associated with poorer cognitive performance, suggesting that disruption of lithium homeostasis may occur early during disease progression [56].

In mouse models of AD and normal aging, dietary lithium deficiency resulted in increased amyloid-β deposition, tau hyperphosphorylation, microglial activation, synaptic and myelin loss, and cognitive impairment, changes that were partly mediated by increased GSK-3β activity. Importantly, restoration of lithium levels prevented many of these pathological and behavioral alterations, supporting the concept that the maintenance of lithium homeostasis may contribute to neuroprotection and that lithium supplementation could represent a potential disease-modifying strategy in neurodegenerative disorders [56].

## 5. The Potential Role of Lithium in Glaucoma

**Figure 1:**
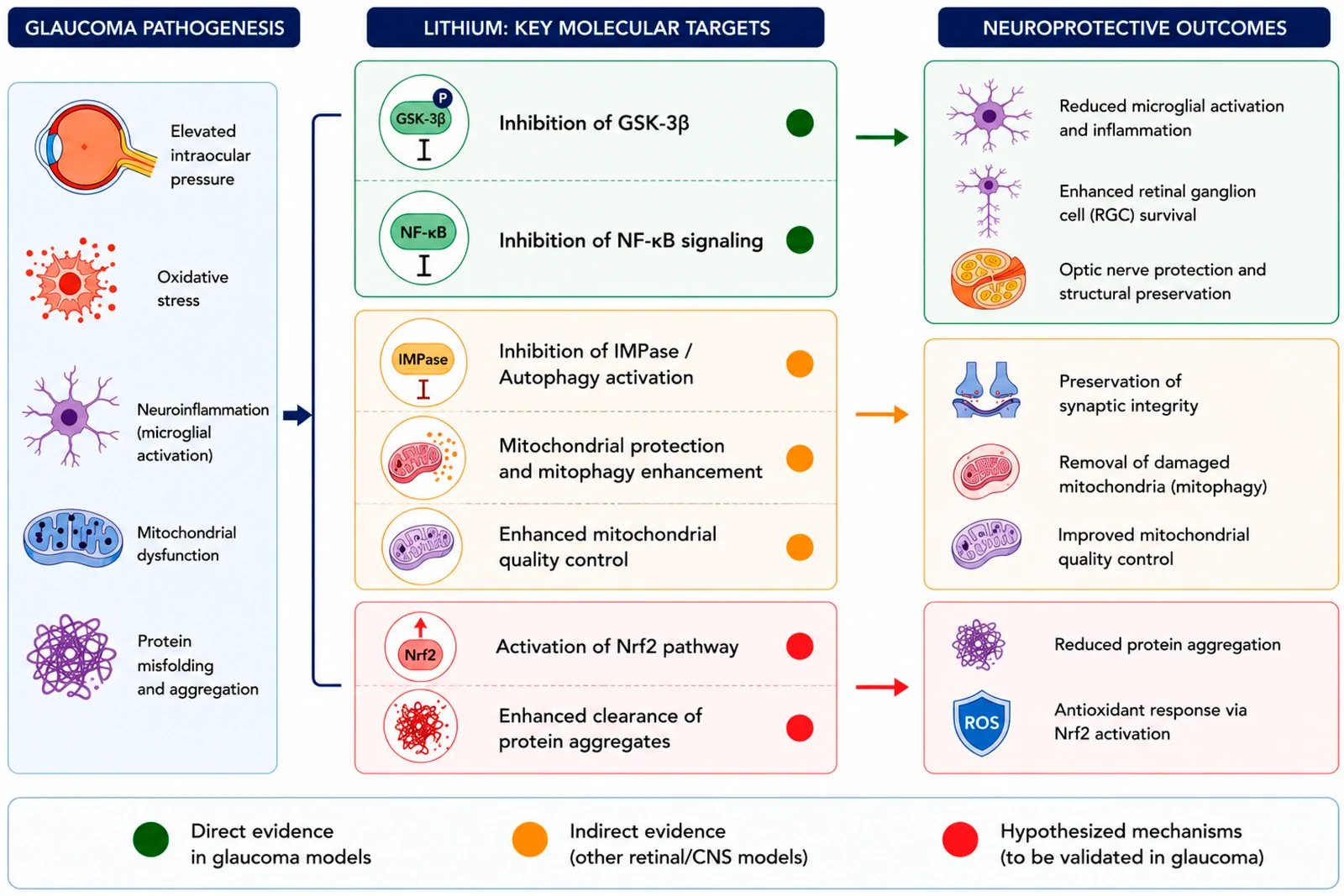
An evidence-based overview of the proposed neuroprotective mechanisms of lithium in glaucoma. Lithium may exert neuroprotective effects through the modulation of multiple pathways involved in glaucomatous neurodegeneration, including GSK-3β inhibition, suppression of neuroinflammatory signaling, regulation of autophagy and mitochondrial homeostasis and antioxidant responses. The color-coded scheme indicates the current level of evidence supporting each mechanism: green, direct evidence obtained in glaucoma models; orange, indirect evidence derived from other retinal or neurodegenerative disease models; red, hypothesized mechanisms that remain to be experimentally validated in glaucoma.

In recent years, lithium has garnered growing interest as a potential neuroprotective compound in glaucoma, although only a limited number of studies have directly investigated its effects on RGC survival and ON degeneration [64]. Nevertheless, the convergence of molecular targets of lithium and the pathophysiological mechanisms underlying glaucoma strongly supports further investigation (Figure 1).

At the cellular level, lithium exerts neuroprotective effects through multiple complementary mechanisms. It counteracts oxidative stress, reduces neuronal hyperexcitability, enhances the expression of neurotrophic and anti-apoptotic factors, and modulates key intracellular signaling cascades involved in cell survival and death [55].

Oxidative stress is a well-established contributor to RGC degeneration in glaucoma, driven by mitochondrial dysfunction, ischemia–reperfusion injury, and chronic neuroinflammation. Lithium has been shown to reduces the production of ROS, enhances mitochondrial membrane stability, preserves mitochondrial respiratory chain function, and prevents mitochondrial permeability transition, thereby limiting oxidative stress-induced cellular injury. These effects are partly mediated through inhibition of GSK-3β, modulation of calcium homeostasis, upregulation of anti-apoptotic proteins such as Bcl-2, and enhancement of endogenous antioxidant defenses. By preserving mitochondrial integrity and cellular energy metabolism, lithium may protect vulnerable neuronal populations from apoptosis and progressive neurodegeneration, mechanisms that are also implicated in glaucomatous optic neuropathy [62].

Inhibition of GSK-3β by lithium promotes stabilization of β-catenin and activation of WNT-dependent transcriptional programs that favor neuronal survival and axonal repair. Experimental studies have demonstrated that lithium enhances axonal regeneration and neuronal resilience at therapeutic concentrations commonly used for bipolar disorder management, typically ranging from 0.5 to 1.2 mM, particularly following long-term administration [24,63,64].

In addition to its effects on GSK-3β signaling, lithium confers protection against several stressors that are highly relevant to glaucomatous neurodegeneration. Experimental evidence indicates that lithium attenuates glutamate-induced excitotoxicity, a major contributor to RGCs death in glaucoma. Elevated extracellular glutamate leads to excessive activation of NMDA (N-methyl-D-aspartate) receptors, calcium overload, and activation of apoptotic cascades [64]. Lithium reduces glutamatergic hyperexcitability and enhances neuronal tolerance to excitotoxic insults, thereby preserving RGCs viability [62].

Furthermore, lithium protects neurons from deprivation of neurotrophic factors, such as BDNF, which are essential for RGC survival and whose retrograde transport is impaired in glaucoma [64].

A key component of lithium’s neuroprotective action is the upregulation of Bcl-2, a critical anti-apoptotic protein that regulates mitochondrial integrity and neuronal survival. Bcl-2 plays a pivotal role in preventing cytochrome c release and caspase activation, thereby inhibiting programmed cell death. In models of neuronal injury, lithium-induced Bcl-2 upregulation has been associated with enhanced axonal repair and improved neuronal survival. In the context of glaucoma, increased Bcl-2 expression may counteract apoptosis of RGCs exposed to chronic stressors such as elevated IOP, ischemia, and inflammation [63].

Lithium also activates the phosphoinositide 3-kinase (PI3K)/Akt signaling pathway, further reinforcing its anti-apoptotic effects. Through this pathway, lithium suppresses downstream apoptotic signaling and contributes to long-term RGCs preservation [64,65]. Consistent with these findings, lithium has been shown to reduce the expression of Bax (Bcl-2-associated X protein), a pro-apoptotic member of the Bcl-2 family, thereby shifting the balance toward cell survival and preventing RGC damage [66].

Experimental studies have shown that lithium is able to induce autophagy, this effect is primarily mediated through the inhibition of inositol monophosphatase (IMPase), resulting in reduced inositol and inositol-1,4,5-trisphosphate (IP3) levels and activation of an mTOR-independent autophagic pathway [67].

Preclinical studies across multiple neurodegenerative models suggest that modulation of the autophagic pathway may represent a key mechanism underlying the neuroprotective effects of lithium [68,69,70,71]. Indeed lithium-induced autophagy has been associated with reduced protein aggregation [72], attenuation of neuroinflammation [73], removal of damaged mitochondria [70], the preservation of synaptic integrity, and improved neuronal survival [56,70].

The potential relevance of autophagy induction in glaucoma is supported by growing evidence indicating that modulation of the autophagic machinery influences RCGs survival under conditions of ocular stress [45,50,74,75,76]. Experimental studies have shown that elevated IOP, ischemia–reperfusion injury, oxidative stress, and neuroinflammation profoundly affect autophagic flux within retinal tissues [44,49,77]. Under these conditions, preservation or enhancement of autophagic flux appears to support RGC survival, whereas autophagic dysfunction may contribute to neuronal vulnerability and disease progression [49,74,78]. Therefore, lithium-induced autophagy may contribute to RGC survival and neuroprotection in glaucoma.

Beyond apoptosis and autophagy regulation, lithium has been implicated in promoting neuronal plasticity and repair mechanisms. It enhances neurite outgrowth, upregulates the transcription factor cAMP response element-binding protein (CREB), and improves DNA repair pathways in primary cultured retinal neurons [55]. CREB activation is particularly relevant in glaucoma, as it regulates genes involved in neuronal survival, synaptic plasticity, and stress resistance. By supporting these adaptive responses, lithium may contribute not only to neuroprotection but also to functional recovery of damaged RGCs.

Lithium may also exert neuroprotective effects through modulation of endoplasmic reticulum (ER) stress pathways, which has been implicated in glaucomatous neurodegeneration. In particular, lithium chloride has been shown to activate the protein kinase R-like endoplasmic reticulum kinase (PERK) pathway, an early adaptive response that promotes cellular survival under stress conditions. In experimental models of glaucoma, PERK activation following lithium treatment was accompanied by reduced expression of Rho-associated kinases (ROCK-1 and ROCK-2), key mediators of cytoskeletal dysregulation and neuronal injury. These molecular changes were further associated with a significant reduction in IOP after chronic administration. Collectively, these findings indicate that lithium may contribute to both neuroprotection and IOP regulation through coordinated modulation of ER stress and ROCK signaling pathways [79].

Despite the compelling preclinical evidence supporting lithium’s neuroprotective potential, several important gaps remain. Clinical evidence on lithium use in individuals with glaucoma remains limited. To date, no prospective clinical trials have directly assessed glaucoma-related outcomes in patients receiving lithium for psychiatric disorders, despite the frequent coexistence of bipolar disorder and age-related ocular disease in routine practice.

Available epidemiological data are nevertheless reassuring. In a nationwide Danish registry analysis, Hajek et al. reported that lithium therapy was not associated with an increased risk of glaucoma and was instead linked to a modest reduction in incident glaucoma after adjustment for relevant confounders [55]. Although these observational findings cannot establish a neuroprotective effect or demonstrate slower disease progression, they support the ocular safety of long-term lithium exposure and align with experimental evidence suggesting the neuroprotective effects of lithium on RGCs.

Accordingly, patients with glaucoma should not be systematically excluded from future clinical trials evaluating lithium as a potential neuroprotective agent. Dedicated prospective studies are required to determine whether chronic lithium therapy influences RNFL thinning, VF deterioration, or other structural and functional biomarkers of glaucoma.

## 6. Challenges in Translating Lithium into Treatment for Glaucoma

Clinical and experimental observations indicate that although glaucoma and AD share several pathogenic mechanisms, they remain fundamentally distinct disorders with different anatomical targets, initiating processes, and clinical phenotypes. As a result, evidence supporting lithium’s neuroprotective effects in AD cannot be directly extrapolated to glaucoma. Within this review, findings derived from central nervous system disorders should therefore be interpreted as mechanistic support for biological plausibility rather than as evidence of a proven therapeutic efficacy in glaucoma (Table 1).

### 6.1. Pharmacokinetic, Safety, and Drug Delivery Challenges

The available direct evidence for the effects of lithium in glaucoma is currently based almost entirely on experimental studies in retinal cell cultures and animal models, whereas human clinical data are limited. There are currently no randomized controlled trials evaluating lithium in glaucoma patients.

Furthermore, despite its promising neuroprotective profile, the clinical translation of lithium for glaucoma requires careful consideration of both its pharmacokinetic profile and its systemic safety concerns. Lithium is a small monovalent cation without protein binding, rapid gastrointestinal absorption after oral administration and almost exclusive renal elimination [80]. In clinical practice, its use is limited by a narrow therapeutic index, with recommended serum concentrations generally ranging from 0.6 to 1.2 mmol/L, depending on the clinical indication and treatment phase [81]. Concentrations above the therapeutic window are associated with a marked increase in the risk of toxicity, making therapeutic drug monitoring mandatory. Therefore, systemic lithium therapy requires regular assessment of serum lithium levels, renal function, thyroid function, electrolytes and calcium metabolism [82]. This is particularly relevant because chronic lithium exposure may induce nephrogenic diabetes insipidus, renal tubular dysfunction, hypothyroidism, hyperparathyroidism, tremor, weight gain and, in predisposed patients, cardiac conduction abnormalities [83,84,85].

Lithium clearance and therefore its serum levels are strongly influenced by renal function and numerous drug interactions. Medications commonly prescribed in older adults, including thiazide diuretics, angiotensin-converting enzyme inhibitors, angiotensin receptor blockers, and non-steroidal anti-inflammatory drugs, may substantially increase serum lithium concentrations and predispose patients to toxicity [86]. These considerations are particularly relevant in glaucoma, a disease that predominantly affects older adults who frequently present with multimorbidity, polypharmacy, and age-related declines in renal function.

A major barrier to the clinical translation of lithium for glaucoma is the limited understanding of its ocular pharmacokinetics. Indeed, in contrast to conventional ophthalmic agents, the pharmacokinetic profile of lithium within ocular tissues has not been systematically characterized, and key parameters, including retinal penetration, intraocular distribution, ocular half-life, and clearance, remain largely unknown.

An important issue is whether the concentrations exerting retinal neuroprotection in experimental models are comparable to those achievable in humans. Seminal studies performed on purified RGCs demonstrated that lithium promotes both RGC survival and axonal regeneration at concentrations ranging from 0.5 to 1.2 mM, which closely overlap with the therapeutic serum concentrations commonly targeted in the treatment of mood disorders [63,81].

However, most in vivo studies investigating the neuroprotective effects of lithium on the retina have employed experimental systemic administration paradigms that do not directly reflect clinically standardized treatment used in humans, particularly with respect to the dose regimen and route of administration.

For example, intraperitoneal administration of lithium reduced IOP and modulated PERK/ROCK signaling in a rat model of glaucoma [79] and promoted RGC survival following ON injury through mechanisms involving Bcl-2 and BDNF upregulation [87,88]. Notably, a more recent study demonstrated that a single intravitreal administration of lithium exerted significant neuroprotective effects after ON transection, increasing RGC survival and reducing apoptotic cell death, thereby providing proof-of-concept that local ocular delivery may represent an effective strategy to directly target retinal tissues while minimizing systemic exposure [89].

A further limitation to the translation of lithium therapy in glaucoma patients concerns ocular bioavailability. The blood–retinal barrier, composed of the retinal vascular endothelium and the retinal pigmented epithelium (RPE), restricts the entry of circulating molecules into retinal tissues and explains why systemic administration often fails to generate predictable therapeutic concentrations in the posterior segment [90]. Direct data on lithium distribution within human retinal tissues are extremely limited. Early pharmacokinetic studies demonstrated that, following oral administration, lithium enters the human aqueous humor [91]; however, its kinetics in ocular fluids differ from those observed in serum, indicating that ocular lithium exposure does not directly mirror systemic concentrations.

Local ocular delivery of lithium represents an appealing therapeutic strategy for either mitigating systemic risks and maximize retinal bioavailability. Intravitreal administration is the most direct approach and has already been explored experimentally for lithium-induced ON neuroprotection [89]. However, repeated intravitreal injections are invasive and may be associated with complications, including endophthalmitis, retinal detachment and patient discomfort. Alternative delivery systems may represent promising strategies to prolong retinal drug exposure while minimizing systemic absorption [92]. Nanoparticle-based platforms have been shown to improve drug delivery to the posterior segment and to increase drug retention within retinal tissues, whereas liposomal formulations can enhance the intravitreal residence time and ocular bioavailability of therapeutic agents [93,94]. In addition, hydrogel-based delivery systems and sustained-release intravitreal implants offer the possibility of maintaining therapeutic drug concentrations within the vitreoretinal compartment over extended periods of time, thereby reducing the frequency of administration and potentially improving therapeutic efficacy and patient compliance [92,93]. Although lithium-specific sustained-release ocular formulations are not yet well established, these technologies provide a feasible framework for future development of lithium-based ocular neuroprotective therapies.

### 6.2. Clinical Translation: Biomarkers, Patient Selection, and Trial Design

The development of biomarkers, the identification of appropriate patient populations, optimization of treatment timing, and carefully designed clinical trials are key elements for the successful clinical translation of lithium treatment in glaucoma. The identification of sensitive biomarkers capable of detecting neuroprotective responses before irreversible RGC loss and VF deterioration occur is one of the highest priorities. Structural optical coherence tomography (OCT), particularly measurements of RNFL and ganglion cell inner plexiform layer (GCIPL) thickness, has become the gold standard for monitoring glaucomatous neurodegeneration and may serve as objective surrogate markers of RGC preservation [95,96,97]. Optical coherence tomography angiography (OCTA) provides complementary assessment of retinal and ONH microvascular integrity and has emerged as a promising biomarker of disease progression, particularly in normal-tension glaucoma [98,99]. Functional biomarkers, including pattern electroretinography (PERG), photopic negative response (PhNR), and standard automated perimetry, should be integrated with structural imaging since electrophysiological dysfunction often precedes irreversible structural loss [100,101,102]. Beyond imaging, molecular biomarkers measurable in aqueous humor, tear fluid, or peripheral blood, including neurofilament light chain (NfL), inflammatory cytokines, oxidative stress markers, and mediators of the GSK-3β/WNT signaling pathway, may ultimately provide pharmacodynamic indicators of lithium activity and facilitate individualized treatment monitoring [103,104,105].

Equally important will be the identification of patient populations most likely to benefit from lithium therapy. Given the heterogeneous pathophysiology of glaucoma, lithium is unlikely to demonstrate uniform efficacy across all disease phenotypes. Patients exhibiting progressive structural or functional deterioration despite adequately controlled IOP, particularly those with normal-tension glaucoma, may represent ideal candidates because neurodegenerative mechanisms appear to contribute disproportionately to disease progression in these individuals [95,106,107]. Likewise, patients with early-stage glaucoma are expected to derive greater benefit from neuroprotective interventions, as a larger proportion of viable RGCs remains available for functional preservation [108]. Future studies should therefore investigate whether imaging, molecular, or genetic biomarkers, including alterations in GSK-3β activity, inflammatory signaling, mitochondrial function, or autophagy-related pathways, can identify patients with increased responsiveness to lithium-based therapies.

An additional critical translational challenge concerns the definition of the therapeutic window. Neuroprotective interventions are generally believed to be most effective before extensive neuronal loss has occurred, emphasizing the importance of initiating treatment during the earliest detectable stages of glaucomatous damage or documented disease progression [95].

Importantly, lithium should be considered an adjunctive rather than a replacement therapy. Lowering IOP remains the only intervention conclusively proven to delay glaucoma progression [109,110,111]. However, lithium targets several pathogenic mechanisms that are largely independent of IOP, including the inhibition of GSK-3β, modulation of neuroinflammation, preservation of mitochondrial integrity, reduction in oxidative stress, and enhancement in autophagic flux [112,113,114]. Consequently, combining lithium with established IOP-lowering medications, laser trabeculoplasty, or glaucoma surgery may provide additive or synergistic neuroprotective effects by simultaneously addressing both the mechanical and neurodegenerative components of glaucomatous optic neuropathy.

The future implementation of lithium therapy may also benefit substantially from precision medicine approaches. Recent advances in genomics, transcriptomics, proteomics, metabolomics, and multimodal retinal imaging have begun to reveal considerable biological heterogeneity among glaucoma patients [115,116,117]. Interindividual variability in inflammatory pathways, mitochondrial function, oxidative stress responses, autophagy regulation, and GSK-3β/WNT signaling may significantly influence therapeutic responsiveness to lithium. Integration of these multimodal biomarkers with artificial intelligence-based predictive algorithms could enable individualized patient selection, optimize benefit–risk assessment, and facilitate personalized neuroprotective treatment strategies [118,119].

Ultimately, successful translation into clinical practice will depend on rigorously designed randomized controlled trials. Initial phase I/II studies should primarily establish ocular safety, tolerability, pharmacokinetics, and dose optimization, particularly for locally administered or sustained-release formulations. Subsequent efficacy trials should enroll patients demonstrating documented structural or functional progression despite adequately controlled IOP and should incorporate sufficiently long follow-up to detect clinically meaningful neuroprotective effects. Primary endpoints should combine structural biomarkers, including OCT-derived RNFL and GCIPL thickness, with functional assessments such as VF progression, PERG, and PhNR, thereby increasing sensitivity for detecting treatment effects before irreversible vision loss occurs [96,97]. Stratification according to glaucoma subtype, baseline disease severity, progression rate, and molecular biomarker profile may further improve identification of treatment-responsive subgroups, while adaptive trial designs and biomarker-guided enrollment strategies could accelerate clinical development [120].

**Table 1:** A summary of the current evidence supporting lithium as a potential neuroprotective therapy in glaucoma. Direct evidence derives from experimental glaucoma models and limited human observational studies, whereas findings from Alzheimer’s disease and other neurodegenerative disorders provide indirect mechanistic support rather than direct evidence of therapeutic efficacy in glaucoma.

Evidence Source	Representative Findings	Strength of Evidence for Glaucoma	Key References
Experimental glaucoma models	Lithium promotes RGCs survival, enhances axonal regeneration, inhibits GSK-3β, reduces apoptosis, oxidative stress and neuroinflammation.	Direct preclinical evidence	[24,63,79]
Retinal cell culture studies	Lithium promotes RGCs survival and inhibits apoptosis through Bcl-2 upregulation, while enhancing neurite outgrowth and neuronal viability.	Direct mechanistic evidence	[63,121]
Human glaucoma studies	No randomized or prospective clinical trials are available. A single nationwide observational study suggests a possible protective effect of long-term lithium exposure, although the evidence remains inconclusive.	Limited direct clinical evidence	[55]
Alzheimer’s disease experimental models	Lithium inhibits GSK-3β, reduces amyloid-β and tau pathology, restores autophagy, attenuates neuroinflammation, and improves cognitive performance.	Indirect mechanistic evidence	[56,59,67]
Parkinson’s disease and Huntington’s disease models	Lithium enhances autophagy, promotes the clearance of aggregation-prone proteins, preserves mitochondrial function, and improves neuronal survival.	Indirect mechanistic evidence	[69,70]
Human Alzheimer’s disease studies	Epidemiological studies suggest that long-term lithium exposure is associated with a reduced risk of dementia, whereas clinical trials have yielded heterogeneous and inconclusive results.	Indirect clinical evidence	[57,59]

Overall, although substantial experimental evidence supports the neuroprotective potential of lithium, significant translational challenges remain before its incorporation into glaucoma management can be realized. Addressing these challenges will determine whether lithium can ultimately evolve from a promising experimental neuroprotective agent into a safe and effective disease-modifying therapy for glaucoma.

## 7. Conclusions

Lithium emerges as a compelling candidate for neuroprotection in glaucoma, given its ability to target multiple converging pathogenic mechanisms implicated in RGC degeneration. Importantly, lithium’s multimodal activity and pleiotropic effects distinguish it from conventional glaucoma therapies that primarily target IOP holding potential as a disease-modifying agent capable of complementing existing IOP-lowering strategies.

Although several neurodegenerative disorders share pathogenic mechanisms with glaucoma, AD was selected as the principal translational model because it exhibits substantial overlap in molecular neurodegenerative pathways. These shared mechanisms (i.e., GSK-3β dysregulation, oxidative stress, mitochondrial dysfunction, impaired autophagy, neuroinflammation, glutamate excitotoxicity, and apoptotic signaling) make AD a particularly informative framework for exploring the biological plausibility of lithium as a neuroprotective agent in glaucoma.

Although clinical evidence in humans is currently lacking, lithium represents a promising and mechanistically well-supported candidate for neuroprotective intervention in glaucoma. Well-designed preclinical and clinical studies are now needed to confirm its efficacy and safety and to optimize targeted ocular delivery strategies, which may ultimately enable a disease-modifying therapeutic approach that complements IOP lowering by directly preserving RGC and ON integrity.

## Data Availability

No new data were created or analyzed in this study. Data sharing is not applicable to this article.

## Abbreviations

The following abbreviations are used in this manuscript:

AD	Alzheimer’s disease
Apo	Apolipoprotein
APP	Amyloid precursor protein
Aβ	β-amyloid
Bax	Bcl-2-associated X protein
Bcl-2	B-cell lymphoma 2
BDNF	Brain-derived neurotrophic factor
CREB	cAMP response element-binding protein
ECM	Extracellular matrix
ER	Endoplasmic reticulum
GSK-3β	Glycogen synthase kinase-3 beta
HD	Huntington’s disease
IMPase	Inositol monophosphatase
IOP	Intraocular pressure
IP3	Inositol-1,4,5-trisphosphate
MCI	Mild cognitive impairment
Nf-kB	Nuclear factor kappa-light-chain-enhancer of activated B cells
NMDA	N-methyl-D-aspartate
ON	Optic nerve
ONH	Optic nerve head
PD	Parkinson’s disease
PERK	Protein kinase R-like endoplasmic reticulum kinase
PI3K	Phosphoinositide 3-kinase
POAG	Primary open-angle glaucoma
pTau	Phosphorylated tau
RGCs	Retinal ganglion cells
RNFL	Retinal Nerve Fiber Layer
ROCK	Rho-associated kinases
ROS	Reactive oxygen species
SNc	Substantia nigra pars compacta
TM	Trabecular meshwork
VF	Visual field
VTA	Ventral tegmental area
